# The effect of providing skilled birth attendance and emergency obstetric care in preventing stillbirths

**DOI:** 10.1186/1471-2458-11-S3-S7

**Published:** 2011-04-13

**Authors:** Mohammad Yawar Yakoob, Mahrukh Ayesha Ali, Mohammad Usman Ali, Aamer Imdad, Joy E Lawn, Nynke Van Den Broek, Zulfiqar A Bhutta

**Affiliations:** 1 Division of Women and Child Health, The Aga Khan University, Stadium Road, P.O. Box 3500, Karachi-74800, Pakistan; 2 Saving Newborn Lives/Save the Children-US, Cape Town, South Africa; 3 Liverpool School of Tropical Medicine, Liverpool, UK

## Abstract

**Background:**

Of the global burden of 2.6 million stillbirths, around 1.2 million occur during labour i.e. are intrapartum deaths. In low-/middle-income countries, a significant proportion of women give birth at home, usually in the absence of a skilled birth attendant. This review discusses the impact of skilled birth attendance (SBA) and the provision of Emergency Obstetric Care (EOC) on stillbirths and perinatal mortality.

**Methods:**

A systematic literature search was performed on PubMed/MEDLINE, Cochrane Database and the WHO regional libraries. Data of all eligible studies were extracted into a standardized Excel sheet containing variables such as participants’ characteristics, sample size, location, setting, blinding, allocation concealment, intervention and control details and limitations. We undertook a meta-analysis of the impact of SBA on stillbirths. Given the paucity of data from randomized trials or robust quasi-experimental designs, we undertook an expert Delphi consultation to determine impact estimates of provision of Basic and Comprehensive EOC on reducing stillbirths if there would be universal coverage (99%).

**Results:**

The literature search yielded 871 hits. A total of 21 studies were selected for data abstraction. Our meta-analysis on community-based skilled birth attendance based on two before-after studies showed a 23% significant reduction in stillbirths (RR = 0.77; 95% CI: 0.69 – 0.85). The overall quality grade of available evidence for this intervention on stillbirths was ‘moderate’. The Delphi process supported the estimated reduction in stillbirths by skilled attendance and experts further suggested that the provision of Basic EOC had the potential to avert intrapartum stillbirths by 45% and with provision of Comprehensive EOC this could be reduced by 75%. These estimates are conservative, consistent with historical trends in maternal and perinatal mortality from both developed and developing countries, and are recommended for inclusion in the Lives Saved Tool (LiST) model.

**Conclusions:**

Both Skilled Birth Attendance and Emergency/or Essential Obstetric Care have the potential to reduce the number of stillbirths seen globally. Further evidence is needed to be able to calculate an effect size.

## Background

Of the estimated global burden of 2.6 million stillbirths per year, around 1.2 million occur during labour i.e. are intrapartum deaths [1]. These are primarily caused by complications arising during labour and childbirth, such as prolonged or obstructed labour or umbilical cord accidents [2,3]. Worldwide 34% of deliveries take place without a skilled birth attendant, translating into 45 million births [4]. Skilled attendance at birth remains particularly low in sub-Saharan Africa and southern Asia. Access to skilled birth care and especially to emergency obstetric care is lowest among the poor, who, therefore, suffer the greatest brunt of maternal and neonatal mortality and morbidity related to complications of childbirth [5]. In high-income countries where women receive good quality skilled intrapartum care, the proportion of stillbirths is less than 10% of all births [3].

To ensure optimal pregnancy outcomes, all women and babies need access to appropriate maternity care in pregnancy, childbirth and after delivery. This includes skilled birth attendance, and provision of basic and emergency obstetric care, for women with complications in pregnancy, childbirth or postpartum. All UN and health care professional organizations strongly advocate for “skilled care at every birth”. The definition of a skilled attendant is “an accredited health professional – such as a midwife, doctor or nurse – who has been educated and trained to proficiency in the skills needed to manage normal (uncomplicated) pregnancies, childbirth and the immediate postnatal period, and in the identification, management and referral of complications in women and newborns” [4].

Basic Emergency (or Essential) Obstetric Care (BEOC) comprises of 7 “signal functions” that include: the use of intravenous/intramuscular antibiotics, intravenous/intramuscular oxytocin, intravenous/intramuscular anticonvulsants, manual removal of retained placenta and removal of retained products of conception(e.g. by Manual Vacuum Aspiration), assisted vaginal delivery and basic newborn resuscitation [6]. Comprehensive Emergency (or Essential) Obstetric Care (CEOC) includes all BEOC signal functions plus Cesarean section and blood transfusion [6].

In this paper, we review available evidence to ascertain the effect of provision of skilled birth attendance as well as basic and emergency obstetric care on stillbirths. The primary purpose of this review was to estimate the effectiveness of these interventions and provide estimates for possible incorporation in the Lives Saved Tool (LiST). This process involved qualitative assessment of available evidence according to GRADE criteria and assessment of quantitative data based on rules developed by Child Health Epidemiology Review Group (CHERG) [7,8]. For more details of this process, see the methods section and CHERG methods paper [8].

## Methods

### Searches

A systematic literature search was performed on PubMed/MEDLINE, Cochrane Database and the WHO regional libraries. A hand search of bibliographies of relevant reviews was also conducted. The search strategy included a combination of the Mesh and free text terms such as ‘Nurse Midwives’, ’Skilled Birth Attendance*’*, Stillbirth and ‘Perinatal Mortality’*.* For emergency obstetric care, a separate search was prepared that used the terms, “obstetric care” or “emergency obstetric” combined with ‘stillbirth’ or “perinatal mortality”. The last date of the search was March 2010.

### Study characteristics and grading

Our primary aim was to select randomized and quasi-randomized trials; however, given the paucity of this evidence, other intervention studies (i.e. before-after) and observational studies were also included. Data of all eligible studies were extracted into a standardized data abstraction sheet [8] containing variables such as participants’ characteristics, sample size, location, setting, blinding, allocation concealment, intervention and control details and limitations [8]. Individual studies were graded based on study design, quality of methods and relevance to study population (middle/lower income countries). Each study was assigned a quality grade of “high” “moderate” “low” or “very low” on the basis of strengths and limitations of the study. Any study with a final grade of ‘very low’ was excluded from the analysis.

The grading of *overall* evidence was based on three components: (1) the volume and consistency of the evidence; (2) the size of the pooled effect and (3) the strength of the statistical evidence reflected in the p-value. A similar grading of ‘high’ ‘moderate’ ‘low’ and ‘very low’ was used for grading the overall evidence indicating the strength of an effect of the intervention on specific health outcome.

### Data synthesis

The primary outcomes were stillbirths and perinatal mortality. Meta-analyses were performed where data were available from more than one study for an outcome. The summary estimates were presented as relative risk (RR) or odd ratios (OR) with 95 % confidence interval (CI). Generic inverse method of meta-analysis was used to pool the data. The assessment of statistical heterogeneity among trials was done by visual inspection i.e. the overlap of the confidence intervals among the studies, and by the Chi square (P-value) of heterogeneity in the meta-analyses. A low P value (less than 0.10) or a large chi-squared statistic relative to its degree of freedom was considered as providing evidence of heterogeneity [9]. The I^2^ values were also looked into and values greater than 50% were taken as substantial heterogeneity. In situations of substantial heterogeneity being present, causes were explored by sensitivity analysis and random effects model were used. Although random models are not a substitute for a thorough investigation of heterogeneity, it takes an ‘average’ effect from all the included studies compared to fixed models that take the exact contribution from the individual studies [9]. All the analyses were performed using Review Manager software version 5 [10].

### Delphi process for establishing expert consensus

Given the paucity of experimental data for these interventions, we sought expert consensus via a standardized Delphi method [8]. The panel invited to participate included experts in maternal and fetal health representing six WHO regions (South Asia, Africa, Western Europe, Eastern Europe, North America, Australia), and included multiple disciplines; international health, obstetrics/gynecology, midwifery, etc. Twenty seven experts agreed to participate in the Delphi process. The questionnaire was developed by MYY, JEL, and ZAB, and refined after several rounds of pilot testing. The questionnaire was sent by email and included the background and aims of the Delphi and estimates of effect that were available from the literature for different scenarios. The median response and range were determined for each question. Consensus was defined a priori as an inter-quartile range in responses of not more than 30% for each question. For those estimates which were at clear variance, clarification was sought from experts and consensus achieved after a maximum two iterations.

## Results

**Figure 1:**
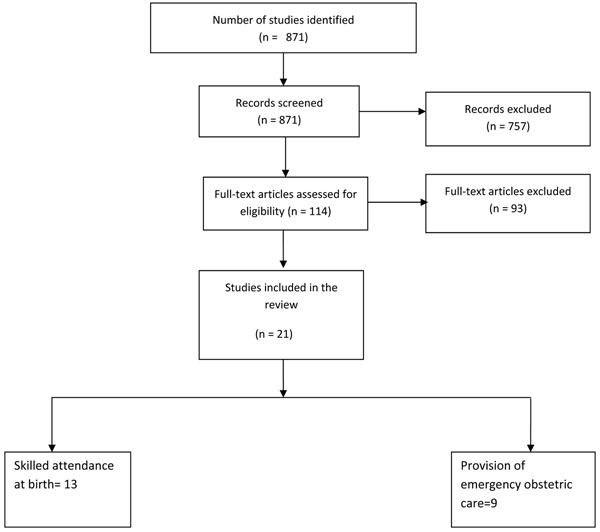
Flow diagram showing identification of studies

The literature search yielded 871 titles (Figure 1). A total of 114 studies were initially identified and screened for eligibility, out of which 4 reviews and 21 studies were finally selected for data abstraction (Additional File 1).

### Role of skilled birth attendance

There are a number of observational studies indicating the impact of skilled birth attendants (midwives, nurses etc.) on perinatal outcomes. Additional File 2 shows the characteristics of studies included in this section. Majority of these studies refer to training or retraining of staff. A study from Sudan, reported a 25% reduction in stillbirths and neonatal deaths with training of the village midwives compared to control [11]. Similar results on training midwives were reported in other studies [12-18]. Ronsmans et al. showed a 24% reduction in stillbirths after the introduction of a safe motherhood program including skilled birth attendant promotion in Matlab district Bangladesh [19-21]. A quasi-experimental study, comparing zones with good access to functioning maternity units (with nurses and midwives) compared to zones with no trained skilled attendants, showed that the stillbirth rate was 22/1000 in the former group compared to 16/1000 in the latter [22]. Provision of skilled attendance at birth in Tanjungsari district in Indonesia [23], showed that the perinatal mortality rate decreased in the intervention district from 50/1000 to 37.4/1000 compared to no decrease in perinatal mortality in the control district of Cisalak.

**Figure 2:**
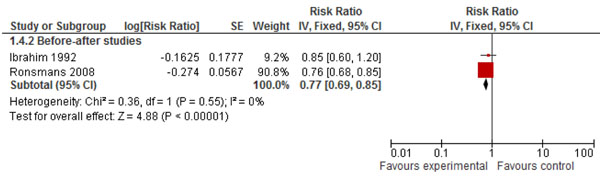
Impact of community-based skilled birth attendance on stillbirths

**Figure 3:**
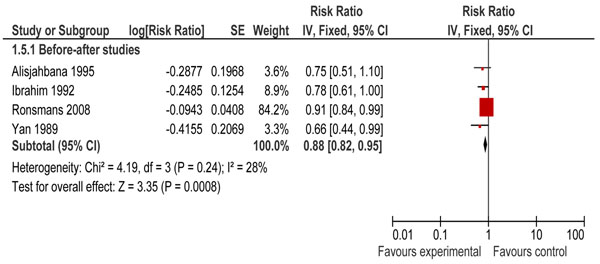
Impact of community-based skilled birth attendance on perinatal mortality

**Figure 4:**
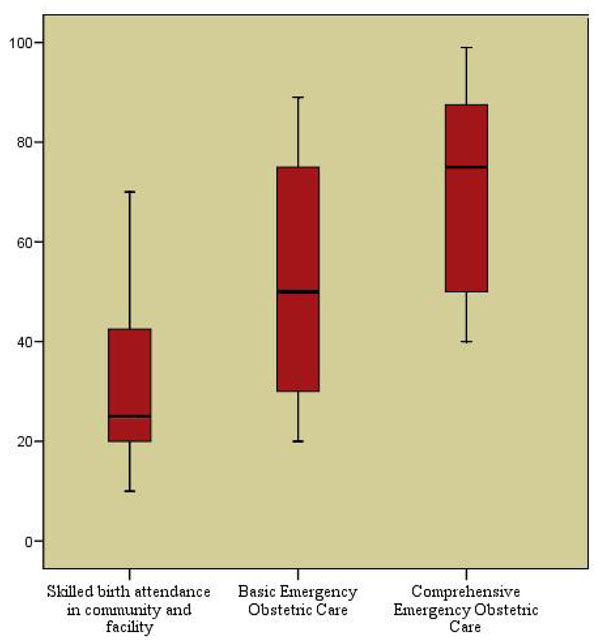
Results of Delphi process for different intervention

Few studies have reported the specific effects of training and supervision of skilled birth attendants in robust experimental designs. There were four before and after studies that reported data on perinatal outcomes [11,19,23,24]. Pooled analysis of two studies [11,19] revealed a 23% significant reduction in stillbirths (RR = 0.77; 95% CI: 0.69 – 0.85) (Figure 2). The combined results of these four studies showed a 12% significant reduction in perinatal mortality (RR = 0.88; 95% CI: 0.82 – 0.95) (Figure 3). The direction of effect in all the included studies was in favor of intervention and there was no significant statistical heterogeneity in the pooled data. The quality of overall evidence was graded as that of ‘moderate’ level, primarily because of a before-after design. Expert opinion using the Delphi method concurred with this impact estimate and suggested a median effect size of 25% reduction in intrapartum stillbirths (interquartile range 20% to 42.5%) for provision of skilled birth attendance.(Figure 4)

### Provision of basic and comprehensive emergency obstetric care

There is a remarkable paucity of good quality data on the impact of provision of the EOC package of care on stillbirths or perinatal mortality. Our review found only low-quality historical and ecologic studies on the subject which are described below. Clearly full RCTs on the subject would be unethical and forthe existing studies the heterogeneity for common comparisons is so great that it precludes meaningful pooled analysis. In the Goldenberg review [39] there was a demonstrable relationship of intrapartum and antepartum stillbirth rates in 51 countries with various measures of obstetric care [25]. There was a decrease of 1.13 stillbirths per 1% increase in delivery by cesarean section as the coverage for this increased from 0 to 9%, but this relationship was not significant. With each 1% increase in cesarean section rates (from 0 to 8%), there was a decrease of 1.6per 1000 births in stillbirths. There was a much smaller decrease in stillbirths as cesarean section rates rose above 8%. This relationship was largely observed in developing countries with overall cesarean section rates of less than 15%. Consistent with these findings analysis by McClure et al. on a larger data set from 188 developed and developing countries provided by the World Health Organization showed that for developing countries, there was a strong decrease in stillbirth rates as the cesarean section rates increased from 0 to about 10%, with little relationship thereafter.[26].

Chigbu and Iloabachie [38] in a prospective controlled study looked at the burden of cesarean section refusal in Nigeria and found that women who refused elective cesarean section had a significantly higher perinatal mortality (34%) versus a matched control group of women who accepted cesarean section (5%), P < 0.001 [27]. Several observational studies provide indirect evidence of the relationship between availability and utilization of emergency obstetric services and intra-partum stillbirths [5,19,28,29].

Given that most of the data was related to observational studies and time trend analyses, to obtain a summary estimate for effectiveness of these interventions for LiST, we conducted an expert Delphi consultation described previously. The Delphi process indicated median estimates of reduction in stillbirths with universal provision of Basic and Comprehensive EOC could avert 45% and 75% of intrapartum stillbirths respectively, although the interquartile ranges for these estimates fell between 30-40% (Figure 4).

## Discussion

Our review highlighted the paucity of systematic analyses of the impact of skilled obstetric care at various levels of the health system on perinatal outcomes including stillbirths. Perhaps the most compelling evidence supporting the link between skilled birth attendance and birth outcomes is the review by Goldenberg et al. [25] who evaluated data from 51 countries and demonstrated a relationship between coverage level with skilled birth attendance and intrapartum stillbirths. They observed a decrease of 0.27 intrapartum stillbirths per 1000 births for each 1% increase in skilled birth attendance from 0 to 54% and a decrease of 0.13 intrapartum stillbirths per 1000 births for coverage with Skilled Birth Attendance between 54% to 100%. McClure et al.[37] in their review on data from 188 developed and developing countries showed that there was no relationship between skilled birth attendance and stillbirths until a coverage threshold of 40% was reached with a nearly linear relationship thereafter.

**Figure 5:**
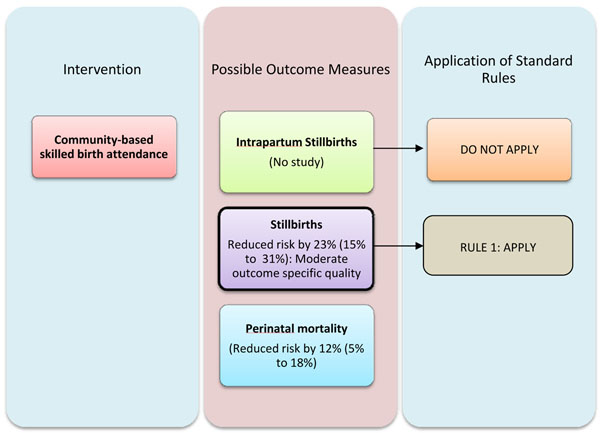
Application of CHERG rules to estimate the effectiveness of skilled attendance at birth to reduce stillbirths

There is universal agreement that deliveries should be conducted by an appropriate skilled birth attendant regardless of place of delivery. Meta-analysis of two observational studies shows a 23% reduction in stillbirths with skilled birth attendance [RR 0.77; 95 % CI 0.69-0.85]. Previous meta-analyses have focused on the effect on perinatal and neonatal mortality. In this paper we have focused on the effect on stillbirths [5]. The quality of evidence for this was however graded as “moderate”. (Table 1)whereas and in Figure 5 we present the application of standardized CHERG rules to the outcome stillbirths and perinatal mortality [8]. Although the Delphi process suggested a slightly higher figure of 25% affect, given that we have direct estimates from the meta-analysis, we would recommend using 23% reduction in the incidence of intrapartum stillbirths with provision of skilled birth attendance for incorporation in the LiST model [8].

**Table 1:** Quality assessment grade table of impact of skilled birth attendance on stillbirth

Quality Assessment						Summary of Findings		
				**Directness**		**No of events**		

**No. of studies (ref)**	**Design**	**Limitations**	**Consistency**	**Generalizability to population of interest**	**Generalizability to intervention of interest**	**Intervention**	**Control**	**Relative Risk (95% CI)**

**Impact of skilled birth attendance on stillbirth: MODERATE outcome specific quality**								

2	Before-after studies	Before-after studies had no control group but only comparison between two time periods	Low heterogeneity. Studies were consistent with both studies showing direction of benefit and out of these two, one was statistically significant	Yes, all studies were in developing countries	yes	Generic inverse variance	Generic inverse variance	0.77 (0.69 - 0.85)

Although the evidence in support of basic and comprehensive emergency obstetric care is compelling, most of the evidence comes from observational studies and most have focused on Cesarean Section which is only one component of Comprehensive emergency obstetric care. The components of the intervention package evaluated in different studies were variable and it was not possible to pool the data. As the primary purpose of this paper was to provide a quantitative estimate for input to LiST, we adopted the Delphi process to generate a point estimate, using CHERG guidelines[8]. This yielded estimated impact figures of 45% and 75% reduction with Basic and Comprehensive EmOC respectively. Two historical reviews by Goldenberg et al. [25] and McClure et al. [26] showed that intrapartum stillbirth rates inversely correlated with an increase in cesarean section rates (from 0 to 10% of deliveries) in developing countries, with little relationship above 10%. An important point to note was that in most of the available studies, outcomes were also reported in terms of reduction in maternal mortality. It is well established that maternal mortality is a risk factor for increased perinatal mortality [30,31]. A report from Sweden by Hoberg and Joelsson [32] has shown that a 15 % increase in institutional deliveries gives a maternal mortality decrease of 35 % while with 99 % coverage there was reduction of 85 %. Similar data have been reported from Malaysia and Sri Lanka [33], where compared to baseline, an average increase in institutional delivery rates by 62 % and 55 % respectively, lead to reduction of maternal mortality by 97 % and 98 % respectively. This suggests that the estimated 45% and 75% reduction in stillbirths attributed to ensuring Basic and Comprehensive EOC is plausible and relatively conservative. We therefore recommend these two estimates for inclusion in the LiST model. We recommend that future large scale studies of maternal interventions including programs to scale Skilled Birth Attendance and Essential or Emergency Obstetric Care document the effect on birth outcomes including stillbirths so that these estimates can be validated.

### Conclusions/key messages

Meta-analysis of observational studies showed a significant 23% reduction in stillbirths with skilled birth attendance. This estimate has been recommended for inclusion in the LiST model.

Basic Emergency (or Essential) Obstetric Care is comprised of 7 “signal functions”: the use of intravenous/intramuscular antibiotics, intravenous/intramuscular oxytocics, intravenous/intramuscular anticonvulsants, manual removal of retained placenta, removal of retained products of conception (e.g. by Manual Vacuum Aspiration), and assisted vaginal delivery. Based on opinion from experts in the field, this intervention could avert 45% of stillbirths.

Comprehensive Emergency (or Essential) Obstetric Care is comprised of 9 signal functions (as under 2 plus cesarean section and blood transfusion). A Delphi process based on expert opinion from professionals in the field suggested that this intervention could avert 75% of stillbirths.

## Competing interests

The authors declare no conflicts of interest.

## Authors’ contributions

Professor Zulfiqar A Bhutta developed the review parameters and secured support. Drs Yawar Yakoob, Mahrukh Ali and Usman Ali undertook the literature search, data extraction and analysis under the supervision of Professor Bhutta. Drs Nynke van den Broek and Aamer Imdad also contributed to the manuscript writing process. Dr. Zulfiqar A. Bhutta gave advice in all the aspects of the project and was the overall supervisor.

## Supplementary Material

Additional File 1 Data extraction sheet for studies included in the review Click here for file

Additional File 2 Characteristics of included studies Click here for file
