# Supplementary material for: The effect of providing skilled birth attendance and emergency obstetric care in preventing stillbirths
Source: BMC Public Health. 2011 Apr 13;11(Suppl 3):S7. doi: 10.1186/1471-2458-11-S3-S7 (PMC3231913; doi:10.1186/1471-2458-11-S3-S7)
Supplement: Additional File 2 — Characteristics of included studies [file 1471-2458-11-S3-S7-S2.docx]

**Additional File 2: Studies on impact of skilled attendance at birth on stillbirths and perinatal mortality**

| **Author** | **Years of study** | **Country** | **Setting** | **Study Design** | **Primary Intervention** | **Concurrent Interventions** | **Intervention Coverage** | **SB/PMR in intervention** | **SB/PMR in control** | **Effect on outcome (95% CI)** |
| --- | --- | --- | --- | --- | --- | --- | --- | --- | --- | --- |
| Alisjahbana 1995 [31] | 1992 - 1993 | Indonesia | Rural West Java | 1.Before-after  2.Quasi-experimental | Comprehensive package of traditional birth attendant (TBA) training, village birthing homes with skilled attendants, capacity building in facilities and strengthening facility-community transport links | None | 86% of women were delivered by TBAs in the intervention district | 1. PMR = 37.4/1000 (44/1176)  2. PMR = 99/2275 | 1. PMR = 50.0/1000 (55/1099)  2. PMR = 37/1000 | 1. RR = 0.75 (0.51 – 1.10)  2. RR = 1.18 (0.81 – 1.70) |
| Alto 1991 [30] | 1981 through 1989 | Papua New Guinea | Nipa district of Southern Highlands Province (the Angal Heneng language group) | Quasi-experimental | 32 Angal Heneng women received instruction as village midwives at Nipa Health Centre | Delivery kits also provided | In 1987, 98% of Angal Heneng women attended antenatal clinic at least once during pregnancy | SB rate = 11.2/1000 | SB rate = 16.3/1000 | RR = 0.69 (0.32 – 1.46) |
| Andersson 2000 [32] | 1831 - 1899 | Sweden | 18 parishes in northern Sweden | Before-after | 1829: midwives were licensed to use forceps, sharp hooks and perforators | 1881: antiseptic techniques were introduced | 73.4% of deliveries being midwife assisted at endline (last decade of the century) compared to 43.7% at baseline (1871 – 1880) | Not mentioned | Not mentioned | 1.Town and sawmill area: PMR = 0.75 (0.66 – 0.84)  2.Farming areas PMR = 0.79 (0.72 – 0.87) |
| Gloyd 2001 [20] | 1987 - 1993 | Mozambique | Rural Manica province | Quasi-experimental | The intervention group composed of villages or towns served by health facilities with trained female nurses or midwives | None | 22% deliveries by untrained person in intervention group vs. 41% in control | SB rate = 22/1000 births | SB rate = 16/1000 births | RR = 1.38 (0.73 – 2.61) |
| Ibrahim 1992 [25] | 1985-1988 | Khartoum, Sudan | Rural, 91% home delivery | Before-after | Training and upgrading of skills of village midwives (antenatal care, monitoring in labour) | Data collection maternal-perinatal outcomes, referral system to hospital | 91% of births delivered by village midwives | SB = 48/2298 | SB = 98/3977 | RR = 0.85 (0.60 – 1.20) |
| Kwast 1996 [22] | 1. 1989 – 1993  2. 1991-1993 | 1.Indonesia  2. Nigeria | 1.Rural West Java  2.Hospital in Bauchi State | 1.Before-after  2. Before-after | 1.Birthing homes (BHs) were introduced with nurse-midwives posted to the project area. TBA training and facility-community transport links established  2. Life saving skills training for midwives and interpersonal communication skills for all providers | 1.None  2. None | 1.85% of births took place at home  2.Midwives performed 52% of all vacuum extractions | 1.PMR = 35.8/1000  2. Intrapartum stillbirths = 1.8% | 1.PMR = 47.7/1000  2. Intrapartum stillbirths = 5.5% |  |
| Montero-Mendoza 2000 [37] | 1996 - 1997 | State of Chiapas, Mexico | Mixed urban/rural | Transversal epidemiological study | Midwife-assisted delivery compared to delivery by a relative, husband or pregnant woman herself | None | 40% birth assistance by a midwife and 3.8% by a relative, husband or pregnant woman herself | Not mentioned in the abstract | Not mentioned in the abstract | OR: 3.31 when birth assistance is provided by the woman's husband or a relative compared to midwife, P < 0.01 |
| PATH 2006 [29] | 2003 - 2006 | Indonesia | Rural Cirebon, West Java | Before-after | Training of Bidan di Desa (village midwives) in neonatal care, including management with birth asphyxia with neonatal resuscitation using tube mask resuscitators | None | 20% deliver in health facilities, SBA rate not reported | SB rate = 6/1000 | SB rate = 8/1000 | RR = 0.75 (0.26 – 2.17) |
| Ronsmans 2008 [26-28] | 1975-2002 | Matlab, Bangladesh | Rural, SBA at home | Before-after | Posting of midwives in villages (antenatal, intrapartum, newborn care) | Strengthening referral systems, Transport to BEMOC | 27% of births attended by SBA in 2001 in ICDDR,B service area | SB rate = 30.4/1000 births | SB rate = 39.8/1000 births | Unadjusted OR = 0.76 (0.68 – 0.84) |
| Sandvik 1993 [34] | 1858 - 1887 | Ytre Nordhordland, Norway | Rural district of western Norway | Intervention study with comparison with national statistics | Appointment of first midwife in 1860 and second one in 1874 | None | Not mentioned | Not mentioned in the abstract | Not mentioned in the abstract | Number of skilled midwives was associated with fewer stillbirths |
| Van Alten 1989 [35] | 1969 - 1983 | Wormerveer, the Netherlands | Hospital and community-based | Observational study of comparison with national figures | Delivery by freestanding midwives either at home, in maternity unit or in the hospital | None | 79% of all parous women booked at the midwives’ practice | PMR = 11.1/1000 | PMR = 14.5/1000 (national figures) |  |
| Van den Broek 2003 [36] |  | Malawi | Rural community in southern Malawi | Descriptive population-based study | Delivery by a trained nurse/midwife compared to a female relative | None | 63% of cases had assistance at delivery by a trained health care worker (doctor or nurse-midwife) | PMR = 21/1000 deliveries | PMR = 47/1000 deliveries | RR = 0.45 (0.27 – 0.74) |
| Walraven 1995 [38] | 1990 | Tanzania | Hospital and community based | Cohort study | Births attended by trained personnel in a dispensary or hospital compared to home births attended only by a relative or a traditional birth attendant (TBA) | None | 19% of women in the study delivered in a dispensary and 28% in the hospital | PMR = 7/202 | PMR = 22/225 | RR = 0.35 (0.15 – 0.81) |
| Yan 1989 [33] | 1980 - 1986 | China | Rural maternal primary health centers in Shunyi county | Before-after | Training of village midwives and doctors. Trained to manage-monitor pregnancy-delivery (blood pressure monitoring, external cephalic version), refer high risk mothers to county hospital. High risk pregnancies screened and booked at county hospital | None | Not mentioned | PMR = 17.6/1000 in 1986 | PMR = 26.7/1000 at baseline |  |
